# The effect of data transformation on low-dimensional integration of single-cell RNA-seq

**DOI:** 10.1186/s12859-024-05788-5

**Published:** 2024-04-30

**Authors:** Youngjun Park, Anne-Christin Hauschild

**Affiliations:** 1 Department of Medical Informatics, University Medical Center Göttingen, Göttingen, Germany; 2 International Max Planck Research Schools for Genome Science, Georg-August-Universität Göttingen, Göttingen, Germany; 3 Campus-Institute Data Science (CIDAS), Georg-August-Universität Göttingen, Göttingen, Germany

**Keywords:** Single-cell sequencing, Dimensionality reduction, Clustering, Cell-type identification

## Abstract

**Background:**

Recent developments in single-cell RNA sequencing have opened up a multitude of possibilities to study tissues at the level of cellular populations. However, the heterogeneity in single-cell sequencing data necessitates appropriate procedures to adjust for technological limitations and various sources of noise when integrating datasets from different studies. While many analysis procedures employ various preprocessing steps, they often overlook the importance of selecting and optimizing the employed data transformation methods.

**Results:**

This work investigates data transformation approaches used in single-cell clustering analysis tools and their effects on batch integration analysis. In particular, we compare 16 transformations and their impact on the low-dimensional representations, aiming to reduce the batch effect and integrate multiple single-cell sequencing data. Our results show that data transformations strongly influence the results of single-cell clustering on low-dimensional data space, such as those generated by UMAP or PCA. Moreover, these changes in low-dimensional space significantly affect trajectory analysis using multiple datasets, as well. However, the performance of the data transformations greatly varies across datasets, and the optimal method was different for each dataset. Additionally, we explored how data transformation impacts the analysis of deep feature encodings using deep neural network-based models, including autoencoder-based models and proto-typical networks. Data transformation also strongly affects the outcome of deep neural network models.

**Conclusions:**

Our findings suggest that the batch effect and noise in integrative analysis are highly influenced by data transformation. Low-dimensional features can integrate different batches well when proper data transformation is applied. Furthermore, we found that the batch mixing score on low-dimensional space can guide the selection of the optimal data transformation. In conclusion, data preprocessing is one of the most crucial analysis steps and needs to be cautiously considered in the integrative analysis of multiple scRNA-seq datasets.

**Supplementary Information:**

The online version contains supplementary material available at 10.1186/s12859-024-05788-5.

## Background

Single-cell RNA sequencing (scRNA-seq) enables a high-resolution view of tissues and organisms. With scRNA-seq, it is now possible to understand heterogeneous cell populations by directly sequencing their transcriptome. However, at the same time, observations on a single-cell level result in a higher noise rate in the data due to technological limitations [1]. Although various single-cell technologies are being developed, it remains impossible to capture all the existing RNAs in the cells since a large proportion of reads are lost during the sequencing preparation steps. The presence of dropouts in scRNA-seq data undermines the precision of gene expression quantification. Various strategies have emerged to handle this challenge from imputation techniques reliant on clustering cells to methodologies leveraging transcriptional regulatory networks and ensemble techniques [2–4]. Thus, proper post-processing of sequencing data is indispensable [5, 6], and various scRNA-seq data analysis tools were developed and introduced during the last decade.

The current practice in single-cell integrative analysis are composed of the following: (1) preprocessing of read-count data, (2) filtering highly variable genes, (3) applying batch integration model, (4) extract features (PCA), (5) clustering on feature space, and (6) visualization with t-SNE or UMAP [7–10]. Additionally, following the recent development of deep neural networks (DNN) in computer science, many DNN models were introduced in the area of bioinformatics and single-cell analysis. In particular, autoencoder-based models have been introduced for single-cell RNA sequencing analysis in the last couple of years. These were used as feature encoders to reduce or filter highly variable genes and represent the data with a relatively small size of latent vectors [11]. Thus, these models represent an alternative for feature extraction from high-dimensional data in addition to classical statistical models using singular vector decomposition [12, 13]. Moreover, generative adversarial networks (GANs)-based models were developed for single-cell data imputations [14, 15] and data augmentation/generation [16], for instance.

Subsequently, a number of benchmark studies introduced a variety of different single-cell analysis tools and evaluated their performance, each focusing on specific steps and challenges in single-cell analysis. For instance, Tran et al. compared 14 different methods for batch-effect correction. Subsequently, they utilized t-SNE and UMAP for the visualization and quality evaluation of the batch correction [17]. According to their evaluation, the top three methods for batch mixing were LIGER [18], Harmony [19], and Seurat v3 [20]. Lytal et al. compared seven different normalization methods for single-cell RNA sequencing data and evaluated these by k-nearest-neighbor cell type classification. They found that the best performing tools vary between the datasets while Linnorm [21] and scran [22] showed consistent results [23]. Li et al. compared four widely used batch correction methods, and found ComBat [24] performed the best according to their criteria [25]. Luecken et al. compared 68 methods and preprocessing combinations using a single-cell dataset including 85 different batches. Their evaluation indicates that, for simple tasks, Harmony [19] is the best choice, and, in more complex tasks, Scanorama [26], and scVI [27] are recommended. Furthermore, they found that deep learning-based models show highly variable performances [28]. Chu et al. compared 28 scRNA-seq de-noising methods in 55 scenarios. They developed a set of pipelines for single-cell processing and compared these for various analysis purposes. Their comprehensive benchmark gives the user a practical view of choices [29].

These benchmark studies highlight the importance of the discussed analysis methods in various tasks. However, the majority of studies neglected the importance of appropriate normalization methods. The preprocessing step for scRNA read counts usually comprises different data transformations. For their benchmark dataset, Luecken et al. used only scran [22] for normalization and log-transformation. However, although this preprocessing step is considered the best choice for single-cell RNA sequencing analysis, up to now, there is no strong evidence that supports the assumption of generalizability across various datasets, and purposes [6]. Moreover, Cole et al. previously reported that there is no one-fits-all solution for every type of single-cell data and pointed out the potential of normalization and data transformation methods for de-noising scRNA-seq datasets [30]. Tian et al. also investigated various analysis pipelines by combining different normalization methods. They also reported that there is not a single best analysis pipeline for all analysis scenarios [31]. In order to get an overview of the data transformation methods used for the analysis of single-cell RNA sequencing data, we reviewed 22 recent studies and found a variety of data transformation statistics, see Table 2, in their preprocessing or data cleaning strategies (Table 1).

**Table 1:** Data transformation methods used in various studies.

Tools	Preprocessing (data transformation used in the study or tool)
scVI [27]	RAW
scLVM [32]	RAW or log-linear fit
scGen [33]	Total $\rightarrow$ Log
MNN [34]	Deconvolution based normalization [35] $\rightarrow$ Log
LIGER [18]	Total $\rightarrow$ l2-norm
scImpute [36]	Total $\rightarrow$ Log10
Scanorama [26]	l2-norm
scIGANs [14]	Minmax
ComBat-seq [37]	RAW
DESC [38]	Total $\rightarrow$ Log $\rightarrow$ Z-score
scMerge [39]	Log $\rightarrow$ Z-score
scDHA [11]	(Log2) $\rightarrow$ Minmax
scVAE [40]	RAW
scGNN [41]	Log
ICAnet [42]	Total $\rightarrow$ Log2
scETM [43]	RAW
iMAP [44]	Log
scBatch [45]	Log, (dataset with ERCC: scPLS [46]) or (Raw $\rightarrow$ ComBat-seq) [37]
Seurat V2 [47], V3 [20]	Total(1e−6) $\rightarrow$ Log $\rightarrow$ Z-score
Harmony [19]	Total(1e−6) $\rightarrow$ Log $\rightarrow$ Z-score
MARS [48]	Total(1e−6) $\rightarrow$ Log $\rightarrow$ min(Z-score, 10)
Benchmark [28]	(scran [22]) $\rightarrow$ Log

Summary of recently published studies for single-cell RNA sequencing data and their data transformation methods. Details about statistics are in Table 2. Preprocessing in parenthesis is an optional step depending on the dataset

For example, log transformation is one of the most common data transformation methods in numerous RNA-sequencing data analysis studies. Another widely used method is the min-max normalization, which is especially favored for deep neural network-based models since these computer vision methods use a 0 to 1 range input array. Moreover, the z-score is another method that has been popular since the microarray era [49]. In comparison, column-wise (cell-wise, or total) normalization is the most widely applied method for single-cell analysis due to technological limitations in single-cell sequencing. This limitation makes it impossible to get an evenly distributed read count in each cell. Consequently, each cell has a different number of total read counts. Therefore, total normalization was introduced to handle this issue [50]. One of the most successful tools for single-cell analysis is Seurat. Thus, many of the studies are using this library [47]. Seurat employs three preprocessing steps: total normalization, log transformation, and Z-score standardization.

**Fig. 1:**
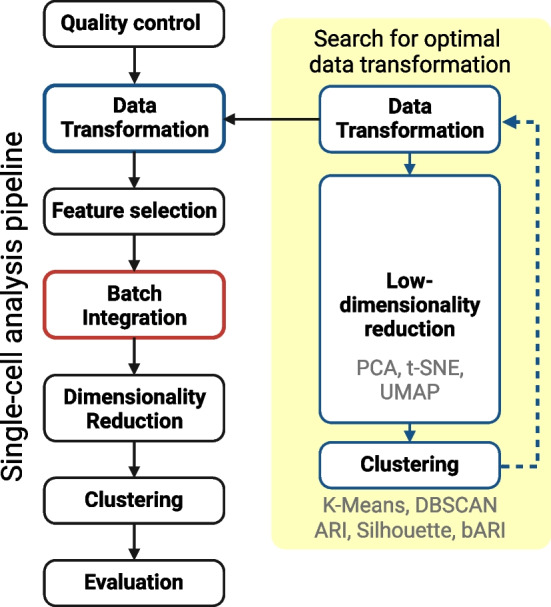
Overview of the proposed low-dimensional analysis workflow for conducting a thorough search for appropriate data transformations. We evaluated the effect of data transformation while integrating different batches of single-cell RNA sequencing data. For that, we tested 16 different data transformations with subsequent dimensionality reduction methods and clustering algorithms and compared their results. This single-cell analysis in conventional practices has feature selection, batch integration, and dimensionality reduction

Previously, Wang et al. compared data transformation methods, including log, raw, and z-score, and two different analysis tools, “sctransform” and “sc3”. In their results, single-cell clustering analysis results were highly dependent on data transformation [51]. However, their work is limited to a few transformation statistics and methods. Furthermore, they solely focused on single-dataset analysis, so batch effects or other noise were not considered. Therefore, in the presented study, we aim to fill this gap and investigate the impact of data transformation methods on both single and multiple-integrated scRNA-seq data analysis. We hypothesize that simple but carefully chosen preprocessing steps can reduce batch effects in the integration of multiple scRNA-seq datasets. Therefore, comparing evaluation results of different datasets and methods without prior optimization and standardization of these preprocessing methods may lead to incomparable outcomes and an unreliable and unfair comparison. To test our hypothesis, we evaluated the impact of a large number of data transformations in integrative scRNA-seq analysis scenarios. Batch effects in heterogeneous datasets are explored using low-dimensional representations, and the results of conventional scRNA-seq analysis are compared (Fig. 1).

## Methods

We tested different combinations of data transformation methods on four different batch effect tasks; (1) single-dataset analysis, (2) multiple datasets analysis, (3) multiple dataset analysis with deep neural networks models, and (4) trajectory analysis with integrated dataset.

*Task 1: Single dataset task* We analyzed individual datasets with a conventional approach and low-dimensional representations with different data transformations. The conventional approach is done by PCA with highly variable gene selection with total normalized and log-transformed data. Subsequently, we evaluate the performance of low-dimensional representation analysis with different data transformation and clustering methods. Details are described in section "Single-cell RNA sequencing data analysis with low-dimensional representation".

*Task 2: Multiple dataset integration task* Multiple datasets analysis was performed on four different subtasks, human pancreas datasets, mouse pancreas datasets, mouse cell atlas datasets, and mouse and human embryo datasets. The aim is to evaluate how much batch effect could be adjusted with simple data transformation methods. Therefore, we will integrate each of the four datasets and employ 16 different data transformation combinations and the dimensionality reduction method, respectively. Finally, the performance of the scRNA-seq clustering results for all subtasks is evaluated as described in section "Single-cell RNA sequencing data analysis with low-dimensional representation". The batch-ARI score is calculated by considering the biological heterogeneity in addition to technological heterogeneity, and the dimensionality reduction results are also visualized on scatter plots with different colors for different mouse strains.

*Task 3: Multiple dataset integration task with DNN models* Next, we set a baseline of the Human Pancreas Dataset from the previous analysis. We evaluated the power of various deep neural network models with the same Human Pancreas Dataset. The Autoencoder, Variational Autoencoder [52], and ProtoTypical Network [53] were tested (Result "Impact of data transformation on the DNN features" section).

*Task 4: Trajectory analysis with integrated dataset* Finally, to generate new insights for the optimal use of the discussed integration approaches, we conducted a follow-up investigation of the downstream scRNA-seq analysis with trajectory analysis with pseudotime. Therefore, we applied various data transformation methods with low-dimensional embedding and clustering to search for proper data transformation. After that, we calculated pseudotime and compared the correlation score with true time points (Result "Subtask 4: Trajectory analysis with integrated dataset" section).

### Dataset description

*Human pancreas dataset* To evaluate severe batch effects in scRNA-seq datasets, integrating multiple datasets from different experiments is a critical step. This dataset allows for a comprehensive analysis of the biological variability and technical artifacts present in the data. We used five public human pancreas datasets (GSE84133, GSE85241, E-MTAB-5061, GSE81608, and GSE83139). These single-cell RNA sequencing datasets and matching annotation information were downloaded. The datasets are available in varying formats, i.e. GSE84133, GSE83139, and E-MTAB-5061 datasets comprise count data, GSE85241 has adjusted count-like data, and only GSE91608 is provided with normalized RPKM. The download scripts are available in the Github repository, and these are based on Hemberg-lab’s work (https://github.com/hemberg-lab/scRNA.seq.datasets). Before analysis, we exclude unclear cell populations in the dataset from the original study, e.g. ’unclassified cell’, ’not applicable’, ’dropped’, or ’no label’. For tasks two and three we integrated all five datasets or batches for batch effect analysis. After integration, the dataset comprises 14,918 cells, 15,628 genes, and 13 cell types. Additionally, we evaluated another human pancreas integration dataset that was analyzed by Zhao et al. [43] (GSE81076, GSE85241, GSE86469, E-MTAB-5061, and GSE84133). This dataset is available with ’SeuratData’ in R [20].

*Mouse pancreas dataset* We used three different datasets for mouse pancreas cells. This mouse pancreas dataset is composed of the Baron Mouse (inDrop) [54], Pancreas from Tabula Muris’s FACS dataset (SMART-Seq2) [55], and Pancreas from Mouse Cell Atlas dataset (microwell-seq) [56]. The Baron mouse and Mouse Cell Atlas datasets and matching annotation information were downloaded via GEO (GSE84133, GSE108097). The Tabula Muris dataset is downloaded from their data portal (https://tabula-muris.ds.czbiohub.org/). Mouse Cell Atlas [56] dataset is available on their website https://bis.zju.edu.cn/MCA/. Cell type and cluster id information for Mouse Cell Atlas is available on the published additional file downloaded from https://ndownloader.figshare.com/files/10760158?private_link=865e694ad06d5857db4b. Each dataset was treated as a different batch. Moreover, we also considered different mouse strains in each dataset. In this case, each mouse strain was treated as a different batch.

For task one, we investigated the batch effect between two mouse strains in the Baron Mouse dataset to compare our approach to a recent benchmark [43]. For task two, we integrated Baron Mouse and Tabula Muris. Labels of the Baron Mouse dataset are converted to make a concordant set with other mouse data: ’activated stellate’ to ’stellate’ and ’quiescent stellate’ to ’stellate’. Labels of the pancreas dataset from Tabula Muris are converted into the same label with Baron Mouse: ’pancreatic A cell’ to ’alpha’, ’type B pancreatic cell’ to ’beta’, ’pancreatic D cell’ to ’delta’, ’pancreatic acinar cell’ to ’acinar’, ’pancreatic ductal cell’ to ’ductal’, and ’pancreatic stellate cell’ to ’stellate’. This integration analysis resulted in 3213 cells and 13,263 genes. The pancreas dataset from Tabula Muris consisted of four mouse strains. Thus, the batch ARI was calculated based on six mouse strain IDs (2 from Baron, and 4 from Tabula Muris). Lastly, we integrated Baron Mouse, Tabula Muris, and Mouse Cell Atlas. For the integration, we filtered cells with available cell labels. For better comparability, we excluded the following non-pancreas-related cell types in MCA data, ’Osteoblast’, ’Myoblast’, ’Cycling cell’, ’Smooth muscle cell’, Stromal cell’, and ’Epithelial cell’. Labels of MCA were also converted to match Baron and TM. As a result, the integration analysis, including the Mouse Cell Atlas datasets, was done with 5171 cells and 12,584 genes.

*Mouse cell dataset* For the mouse cell dataset task, we used the other Tabula Muris datasets except for the pancreas, already used in the previous task. This TM dataset contains data from two different single-cell RNA sequencing protocols, SMART-Seq2 from FACS-sorted cells and 10x Genomics platform with CellRanger. Moreover, each of the datasets contains additional mouse strain information, which was treated as a batch for the analysis. Among the 16 different organs in the TM dataset, we extracted a set of tissues and organs that were sequenced by both sequencing protocols. Namely, Bladder, Kidney, Limbic muscle, Liver, Lung, Mammary Gland, Marrow, Spleen, Thymus, Tongue, and Trachea were selected. In the case of one of the batches having a dominant population of cells (>700k vs <1k), we limit the number of cells in one batch by sampling cells on that batch to prevent bias in the dominant population batch. In the lung and trachea datasets, we sampled 2,000 cells for ’Lung-10X_P8_12’, ’Lung-10X_P8_13’, ’Trachea-10X_P8_14’, and ’Trachea-10X_P8_15’.

*Preimplantation embryo data dataset* The dataset comprising experiments on mouse and human preimplantation embryos with single-cell RNA sequencing is utilized for trajectory analysis on low-dimensional space. The data is obtained from Zenodo (10.5281/zenodo.10669600) [57, 58]. The mouse dataset is an integration of thirteen different studies [59–71]. The human dataset contains six different studies [70, 72–76]. The raw count matrix is used for the evaluation of data transformation methods.

### Single-cell RNA sequencing data analysis with low-dimensional representation

*Individual statistics for data transformation*

We investigated various data transformation methods applied to scRNA-seq data and chose six data transformation methods. These data transformations are applied to the scRNA-seq data to change its distribution to get better training outcomes. Luecken et al. classified data transformation methods into two steps, normalization, and transformation [6]. Because of technological limitations that some cells capture more reads and some cells do not, column-wise normalization has been widely applied (total). Minmax normalization is a straightforward method when multiple datasets are integrated. The standardization step using Z-score is also a popular approach. The log transformation could reduce data skewness. Details are listed in Table 1, where E is the expression profile of the cell and e is each of the genes measured in scRNA-seq.

**Table 2:** Data transformation methods.

RAW	No transformation
Log2	$E = Log_{2}(e+1)$
Total	$E = {\frac{e}{sum(e)}} * 20000$
*l*2-norm	$E = {\frac{e}{\sqrt{ sum(e^2)}}}$
Minmax	$E = {\frac{e - min(e)}{max(e)-min(e)}}$
Z-score	$E= {\frac{e - mean(e)}{std(e)}}$

Where *e* is a vector of gene expression level in a cell

We used six statistics to transform single-cell RNA sequencing data. These statistics are widely used in sequencing data analysis studies

*Combination of data transformation statistics used for the preprocessing benchmark*

Some studies chose an arbitrary data transformation method without further reasoning and fed transformed expression profiles to their complex and novel analysis model. At the same time, we found partial consensus on data transformation methods using three steps: Total$\rightarrow$Log$\rightarrow$Z-score. This is due to the big success of Seurat [47] in the scRNA-seq data transformation: Total$\rightarrow$Log$\rightarrow$Z-score, see Table 2.

For the data transformation benchmark performed in this study, 16 different combinations of normalization are employed using five data transformation methods: Log2-transformation, Total normalization, Minmax normalization, *l*2-normalization, Z-score transformation.

*Dimensionality reduction* Dimensionality reduction is a crucial part of single-cell RNA sequencing analysis. High-dimensional gene expression data is projected into a low-dimensional space to be interpreted. We employed three methods for our analysis. The main analysis is done with PCA and UMAP [77] as a feature extraction technique for clustering. Additionally, we also investigated t-SNE [78]. For t-SNE, we fixed the number of components as two and did not initiate with PCA. For UMAP, we fixed the number of components as two and initiated with the parameter ’spectral’. For K-Means, we searched for the best ARI score amongst varying parameters for the number of clusters, from half of the number of original labels to the number of original labels + 4. The number of iterations was from 20 to a max of 50. For DBSCAN, we searched for the best ARI score during varying eps parameters from 0.5 to 10 with a 0.5 step size. The parameter for a minimum number of samples is fixed to eight. In all parts of the analysis, we used t-SNE, UMAP, K-Means, and DBSCAN [79] from the Python ’scikit-learn’ packages.

For further analysis, many prior processes are applied to the dimensionality reduction method. This prior process is focused on feature extraction. By doing this, PCA, t-SNE, or UMAP could effectively represent the cell-type specific expression features. The feature selection based on highly variable genes is a widely used prior method [6, 80]. Recently, an autoencoder model was employed for variable gene selection [11]. Furthermore, complex DNN models are introduced for feature extraction. We will cover this DNN model-based feature extraction in the next section "Deep neural networks model".

*Clustering evaluation* Clustering algorithms are often applied to latent space generated from the above dimensionality reduction methods for the identification of cell populations. There are various clustering algorithms. For our analysis, we apply the most widely used K-Means clustering, and DBSCAN [79]. Kim et al. reported that the similarity metrics have a critical impact on the single-cell cluster analysis [81]. In our study, we fixed detailed parameters for the clustering algorithm to exclude additional variability. The evaluation of the dimensionality reduction was done with the clustering results using the Adjusted Rand Index (ARI), batch-wise ARI (bARI), and Silhouette score. The ARI is calculated between true cell-type labels and clustering results, and the bARI is calculated between batch ids and clustering results. In the scatter plot, bARI is transformed into 1-bARI and visualized for convenience. The silhouette score is calculated with latent vectors and clustering results. Adjusted Rand Index (ARI), batch-wise ARI (bARI), Silhouette score, and Normalized Mutual Information (NMI) are calculated with Python ’scikit-learn’ packages ’metrics.adjusted_rand_score’, ’metrics.silhouette_score’, and ’metrics.normalized_mutual_info_score’.

*Trajectory analysis* The trajectory conservation score serves as a proxy measure for assessing the preservation of the biological signal. We conducted a trajectory analysis calculated after the integration of multiple datasets about cell differentiation. These trajectories were computed utilizing diffusion pseudotime, as implemented in Scanpy (scanpy.tl.diffmap and sc.tl.dpt). The pseudotime is compared with the time point assigned based on cell types. From ’Zygote’ to E4.5 cells, it is assigned with integer values. The Spearman’s rank correlation between pseudotime and true time point is calculated with (scipy.stats.spearmanr).

The trajectory plots are generated with the partition-based graph abstraction (PAGA) method [82]. PAGA provides an interpretable graph-like map of the data manifold, based on estimating the connectivity of manifold partitions.

### Deep neural networks model

We tested the power of DNN models as a feature extractor for after-dimensionality reduction methods. With this analysis, we incorporate the recent development of DNN model-based tools for single-cell analysis. In this study, we tested five different DNN models, Autoencoder (AE), Variational Autoencoder (VAE), ProtoTypical Network (Proto), and Variational Proto (VProto). We used python and the PyTorch library to implement deep neural network models. The performance of all four types of models was reported on the best model selected from more than 10 training runs using randomly initialized weights.

*Autoencoder and Variational Autoencoder* The basic encoder and decoder block is composed fully connected layer, batch normalization layer, and relu layer. In the AE model, there is one hidden layer sized 1024, and the size of the latent layer is tested with 2, 4, 8, 16, 32, 64, 128, and 256. In the case of VAE, the hidden layers have 1024, 128 sized output vectors, and another fully connected layer produces vectors for $\mu$ and $\sigma$ having 2 to 256 size, similar to AE. The reconstruction loss is calculated with the mean squared error between the original gene expression vector and the reconstructed vector.

*ProtoTypical and variational-ProtoTypical network* ProtoTypical Network is a kind of few-shot learning model having great success in various tasks from computer vision to biomedical analysis [53, 83]. We implemented ProtoTypical Network in two ways, Proto and VProto. The Proto means a general ProtoTypical network that prototypical loss is calculated on latent layer after feature extractor with fully connected layers. The VProto means that prototypical loss is calculated on the latent layer. Specifically, we obtained a latent vector from the reparametrization trick with $\mu$ and $\sigma$ from the feature extractor with fully-connected layers and used it to calculate the prototypical loss. The prototypical loss was euclidean distance on latent space.

*Model training and testing scenario for batch effect analysis* To evaluate the impact of the de-noising power of the data transformation method, we trained the model with only one of five datasets. If the data transformation method has minute power in batch effect adjusting, the model will be easily over-fitted on the training dataset with noise, and evaluation with whole human pancreas datasets would not be good. Furthermore, because the ProtoTypical Network is a supervised learning model [53], utilizing the entire dataset for training and testing simultaneously is nonsense. In this case, we are training the model with one dataset and testing with the other four datasets by comparing cell clusters with the training dataset. For this reason, during the training step in all AE, VAE, Proto, and VProto, we used Baron (Human) dataset and transformed entire human pancreas datasets to visualize and evaluate. With the different sizes of latent vectors, we clustered cells with K-Means and DBSCAN and evaluated their performance in dimensionality reduction and cell population clustering.

## Results

We evaluated 16 different data transformation methods on three tasks with public single-cell datasets, (1) a single dataset analysis task, (2) a multiple datasets integration and batch effect correction task, and (3) a multiple datasets integration task with deep neural networks models (Details in the "Methods" section). Each task was evaluated with regard to the ARI score with its cell-type clustering result on a low-dimensional representation (Methods section "Single-cell RNA sequencing data analysis with low-dimensional representation"). For task one, we investigated the impact of the different data transformations on the individual datasets (section "Impact of data transformation on the analysis of low-dimensional single-cell sequencing data"). Next, we assessed the impact of data transformation on the multiple dataset integration analysis in terms of batch effect correction (section "Impact of data transformation on the multiple dataset integrative task with low-dimensional representation") in task two. We first set a baseline for the evaluation of the cell-type classification tools and compare it to the performance of deep neural network models in feature extraction and batch effect correction (section "Impact of data transformation on the DNN features").

### Impact of data transformation on the analysis of low-dimensional single-cell sequencing data

**Table 3:** Overview of best-performing data transformation methods for UMAP features of single dataset scRNA-seq analysis

Analysis	Dataset	Best	ARI	Worst	ARI
UMAP K-Means	Baron (Human)	Log2	0.789	Total Log Minmax	0.335
	Muraro	Total l2norm	0.956	Total Log2 Z-score	0.276
	Segerstolpe	Z-score	0.645	Log2 Minmax	0.290
	Wang	Total Log2 l2norm	0.856	Total Log2	0.313
	Xin	total l2norm	0.647	Log2	0.040
	Baron (Mouse)	Total Log2 Z-score	0.682	Total Log2	0.107
UMAP DBSCAN	Baron (Human)	l2norm	0.870	Total Log2	0.005
	Muraro	Total l2norm	0.951	Log2 Minmax	0.317
	Segerstolpe	Total Minmax	0.918	Total Log2 Minmax	0.333
	Wang	Total Log2 Z-score	0.853	Total Log2	0.000
	Xin	Total	0.886	Log2	0.000
	Baron (Mouse)	Total	0.847	Total Log2 Minmax	0.000
PCA30 Louvain	Baron (Human)	Log2	0.938	l2norm	0.000
	Muraro	Z-score	0.966	l2norm	0.000
	Segerstolpe	Total Z-score	0.741	l2norm	0.000
	Wang	Total Log2 Z-score	0.957	l2norm	0.000
	Xin	Total Z-score	0.994	l2norm	0.000
	Baron (Mouse)	Z-score	0.919	l2norm	0.000

To demonstrate the impact of data preprocessing on the subsequent analysis, we first extended the single dataset analysis task by Wang2020 [51] and Cole2019 [30]. Our aim is to evaluate various data transformation methods for their suitability as preprocessing for different clustering algorithms on a low-dimensional representation of the data. Therefore, we include K-Means clustering on a UMAP-2D representation, DBSCAN clustering on a UMAP-2D representation, and Louvain clustering on a PCA-30D representation as best-practice approach, details in Methods section "Single-cell RNA sequencing data analysis with low-dimensional representation".

Data transformations that yield the most favorable results for analysis tend to vary from one dataset to another. The comparison between single-cell clustering analysis on low-dimensional representations and best-practice results are shown in Table 3. The Baron (Human) dataset shows the best ARI score with 0.938 using PCA30 combined with Louvain clustering. However, the Segerstolpe dataset shows the best result when analysed with UMAP and DBSCAN clustering resulting in an ARI score of 0.918. This clearly shows that simple data transformation methods can tremendously affect the low-dimensional representation, as demonstrated by an extreme ARI variation ranging between 0.000 and 0.966 pancreas datasets. All visualization results for each dataset are available in a Additional file 1 (see availability of data and materials section).

The Baron (mouse) dataset contains sequencing data from two different mouse strains. In the recent work [43], this dataset was used to evaluate different strain effect correction tests. It is shown as ’MP’ in the table for comparison with the benchmark work (see Additional file 1: Table S1). Furthermore, our results show that the noise from different strains can be mitigated when proper data transformation is applied. We obtained an ARI score of 0.929 from Minmax combined with the t-SNE+DBSCAN analysis (Table 3). In particular, these results indicate that noise and systematic effects from different mouse strains can be mitigated by the application of proper data transformation.

In summary, the results of our single dataset analysis evaluation validated and extended previous findings of Cole et al. and Wang et al. [30, 51]. There is no method that performs equally well on all datasets. A data transformation method that works best for one dataset and a specific analysis pipeline does not necessarily perform well on another.

### Impact of data transformation on the multiple dataset integrative task with low-dimensional representation

To reveal the impact of data preprocessing for de-noising and batch effect correction, we evaluated four subtasks of popular single-cell datasets with the same 16 data transformations and four single-cell RNA analysis pipelines. The four benchmark tasks are human pancreas Dataset, mouse pancreas dataset, mouse cell dataset, and mouse and human embryo datasets.

#### Subtask 1: Human pancreas datasets

**Fig. 2:**
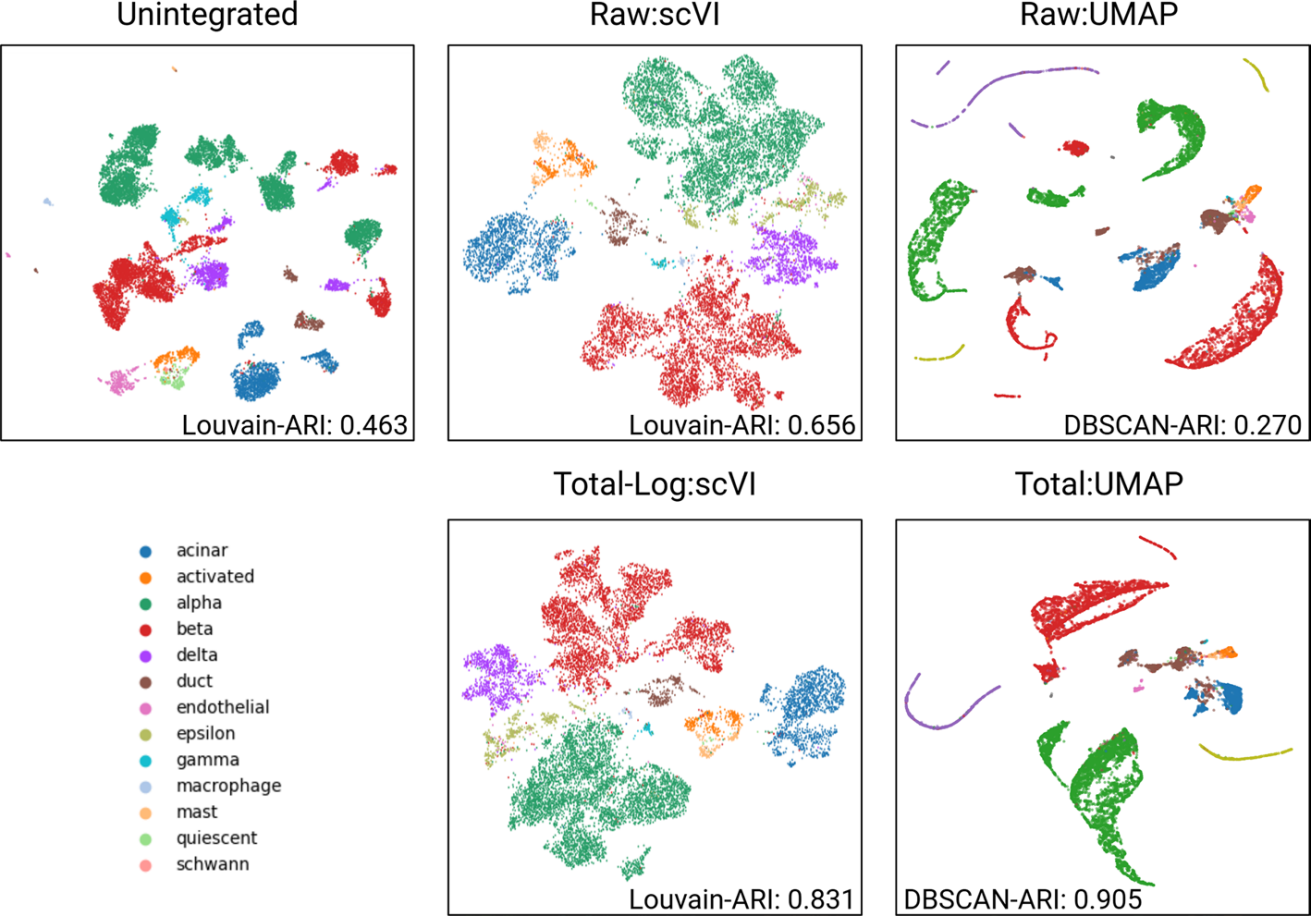
Evaluation of the effects of different data transformation methods on cell-type clustering in low-dimensional representations compared to conventional integration analysis pipelines. For this analysis, we utilized five different single-cell RNA sequencing datasets of human pancreas. Clustering results on low-dimensional representation are compared with results on unintegrated and scVI-integrated datasets. The top-left plot shows the unintegrated data as baseline for batch integration of the five pancreas datasets. It is obtained employing total-Log:HVG (highly variable gene) for preprocessing. The top-center plot corresponds to the result after applying scVI on the raw count data. The result of total-Log:scVI, as shown in the bottom-center plot, is done by applying scVI after normalizing the count matrix with total and log transformation. The plots on top-right and bottom-right show the DBSCAN clustering results on Raw:UMAP and Total:UMAP low-dimensional representations, respectively. The Raw:UMAP 2D representation is based on raw counts data and Total:UMAP 2D representation is obtained on total-normalized data. In both cases, DBSCAN is employed to evaluate cell-type clusters

The selected human pancreas datasets are frequently used for batch correction tools for single-cell sequencing analysis. They consist of five different single-cell RNA sequencing datasets resulting from four different sequencing protocols. They are well-labeled with cell types in the pancreas. We aggregated all five datasets and preprocessed them with a conventional single-cell RNA sequencing analysis procedure using Scanpy library [84]. In the unintegrated dataset, the procedure could not find clear clusters of the same cell types in each batch. Louvain clustering found 21 clusters and scored an ARI score of 0.463 and a 1-bARI score of 0.723 (Fig. 2, top-left). As a goal standard, we applied scVI, which is a widely used conventional approach to integrate different batches. The scVI is able to find better clusters than the procedure on unintegrated data. In the scVI result, we compared raw and the best-practice preprocessing, namely total-log normalization. When raw data was analyzed with scVI, Louvain clustering identified seven clusters with an ARI score of 0.656 and a 1-bARI score of 1.03 (Fig. 2, top-center). When total-log normalization is applied, the scVI features clustered with Louvain found five clusters with an ARI score of 0.831 and 1-bARI score of 1.04 (Fig. 2, bottom-center).

Subsequently, we investigated the effects of batch integration with clustering on low-dimensional representations of the integrated data. All six analysis pipelines, namely PCA:K-means, PCA:DBSCAN, t-SNE:K-Means, t-SNE:DBSCAN, UMAP:K-Means, and UMAP:DBSCAN, are applied to the single-cell datasets integration task. The visualization of the results in Fig. 2 clearly demonstrates which preprocessing pipelines can mitigate the batch effects present in the data. For example, when no transformation is applied prior to the UMAP(2D) representation and DBSCAN clustering (RAW:UMAP, with an ARI score of 0.270), the alpha cells are scattered into three different clusters, and the beta-cell type is split into four different clusters, with another cell type located between them (Fig. 2, top-left). In contrast, when applying Total normalization prior to UMAP(2D) representation, the DBSCAN clustering identified 9 clusters with an ARI score of 0.905 (Fig. 2, bottom-right). More importantly, in the resulting clustering, the alpha-cell type is well clustered. While the beta-cell type is still split, the clusters are located close to each other.

In comparison, on the PCA(2D) representations the best ARI score of 0.746 is achieved when Total:Z-score is applied, identifying 18 clusters. It is possible to identify 12 clusters with an ARI score of 0.822 when Total-*l2*-norm is applied on t-SNE representation. Although, it is commonly known that t-SNE results in a distorted space intended for visualization purposes and it is often controversial to perform post-analysis on t-SNE(2D) representation, it shows surprisingly decent results. All visualized results are available in a Additional file 1 (see Methods section 5.4).

The recent work by Zhao et al. additionally conducted a benchmark analysis using a different composition of human pancreas datasets [43]. Therefore, we compared the performance of low-dimensional approaches to state-of-the-art methods benchmarked in that work. Notably, the result of DBSCAN clustering on the Total:UMAP representation achieved an ARI score of 0.848 in the mouse pancreas dataset and an ARI score of 0.725 in human pancreas dataset (Additional file 1: Table S1). The visualized result is available in Additional file 1: Figure S1. The Louvain clustering showed lower performance than DBSCAN on low-dimensional representation with ARI score of 0.624. This low-dimensional approach was not able to outperform state-of-the-art methods in human pancreas dataset (ARI score of 0.761 with l2-norm:UMAP and 0.955 with Harmony). However, we see comparable results between the state-of-the-art method and our low-dimensional representation methods on the mouse pancreas data (ARI score of 0.929 with Minmax:t-SNE and 0.969 with Harmony).

#### Subtask 2: Mouse pancreas datasets

For the evaluation of the mouse pancreas task, we used three mouse pancreas data sets, Baron (Mouse) [54], Tabula Muris [55], and Mouse Cell Atlas [56]. At first, we integrated and analyzed the Baron and TM pancreas datasets. For the combined application of t-SNE and DBSCAN, Total data transformation was the best normalization method presenting good batch effect correction performance with an ARI score of 0.865. In comparison to the human pancreas dataset, there were multiple choices of the data transformation that performed equally well, *l*2-norm, Minmax, and Z-score. Log2$\rightarrow$Minmax or Total$\rightarrow$Log2 methods showed the worst performance. The latent representation of the original data was clearly separated based on the batch labels. However, Total or the other data transformation method above was able to mitigate the batch effect resulting in a good cluster representation. In the UMAP and DBSCAN results, Total also showed the best batch effect removal performance with an ARI score of 0.842 and a Silhouette score of 0.639. Similarly, for the combination of t-SNE and DBSCAN, Minmax, *l*2-norm, and Z-score methods showed good performance in batch correction.

To further evaluate the impact of preprocessing on the analysis pipelines, we integrated another mouse pancreas dataset from MCA. In particular, we challenged it with an MCA pancreas dataset that does not have a similar set of labels for pancreas-specific cell types. Instead, cell types are aggregated into one label, endocrine cells. Thus, the ARI and bARI scores calculated with these labels are not fully comparable with the previous performance of the batch effect correction. Nevertheless, we could observe that the endocrine cells of the MCA dataset are well spread on alpha, beta, and delta clusters. Moreover, batch effect correction through data transformation can be observed for the epithelial cell type. In most preprocessing, epithelial cells were not grouped into clear cell clusters. However, in the case of Total$\rightarrow$Minmax, they are closely located to each other. All visualized results are available in a Additional file 1 (see see availability of data and materials section)

#### Subtask 3: Mouse cells datasets

**Fig. 3:**
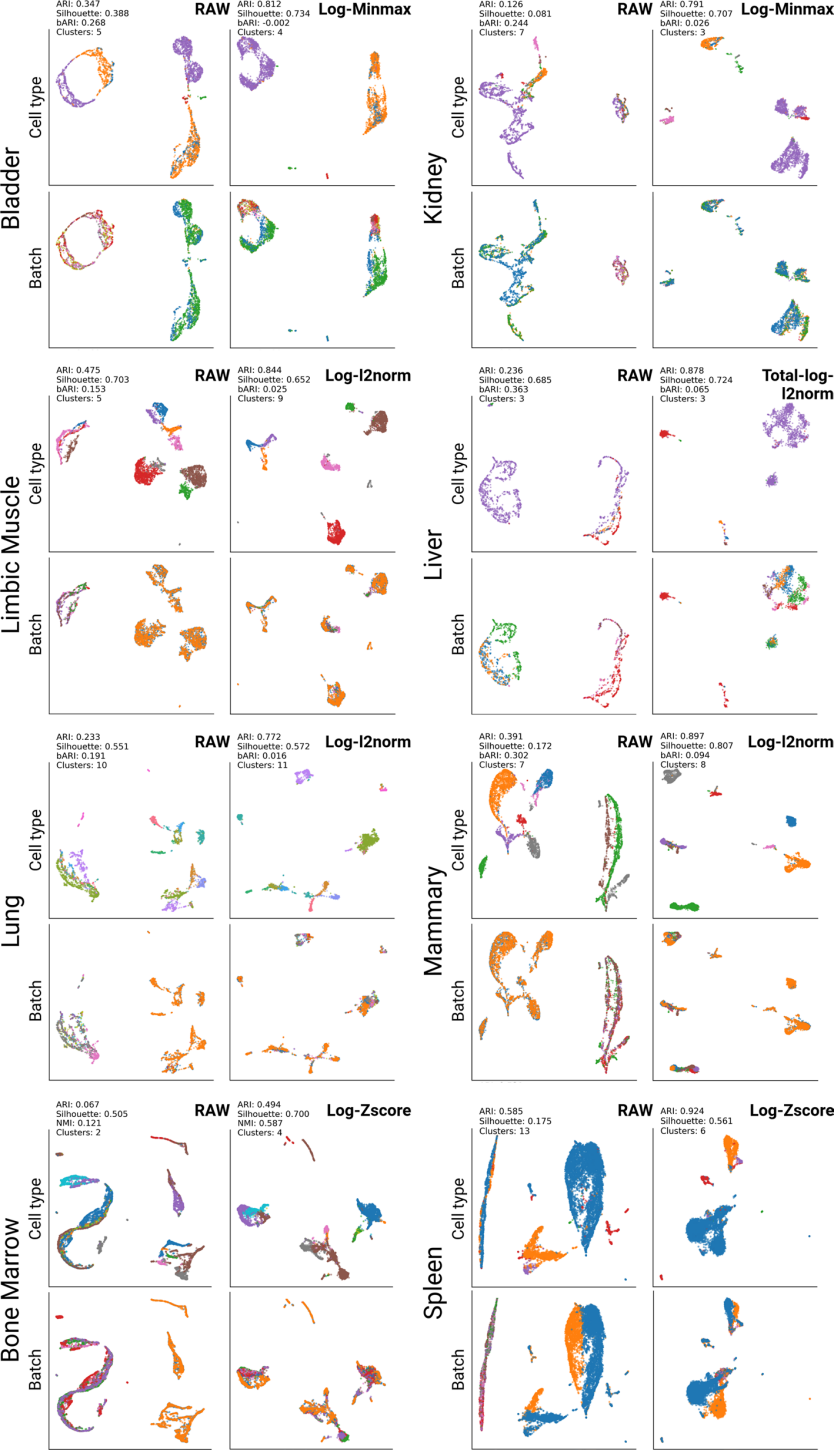

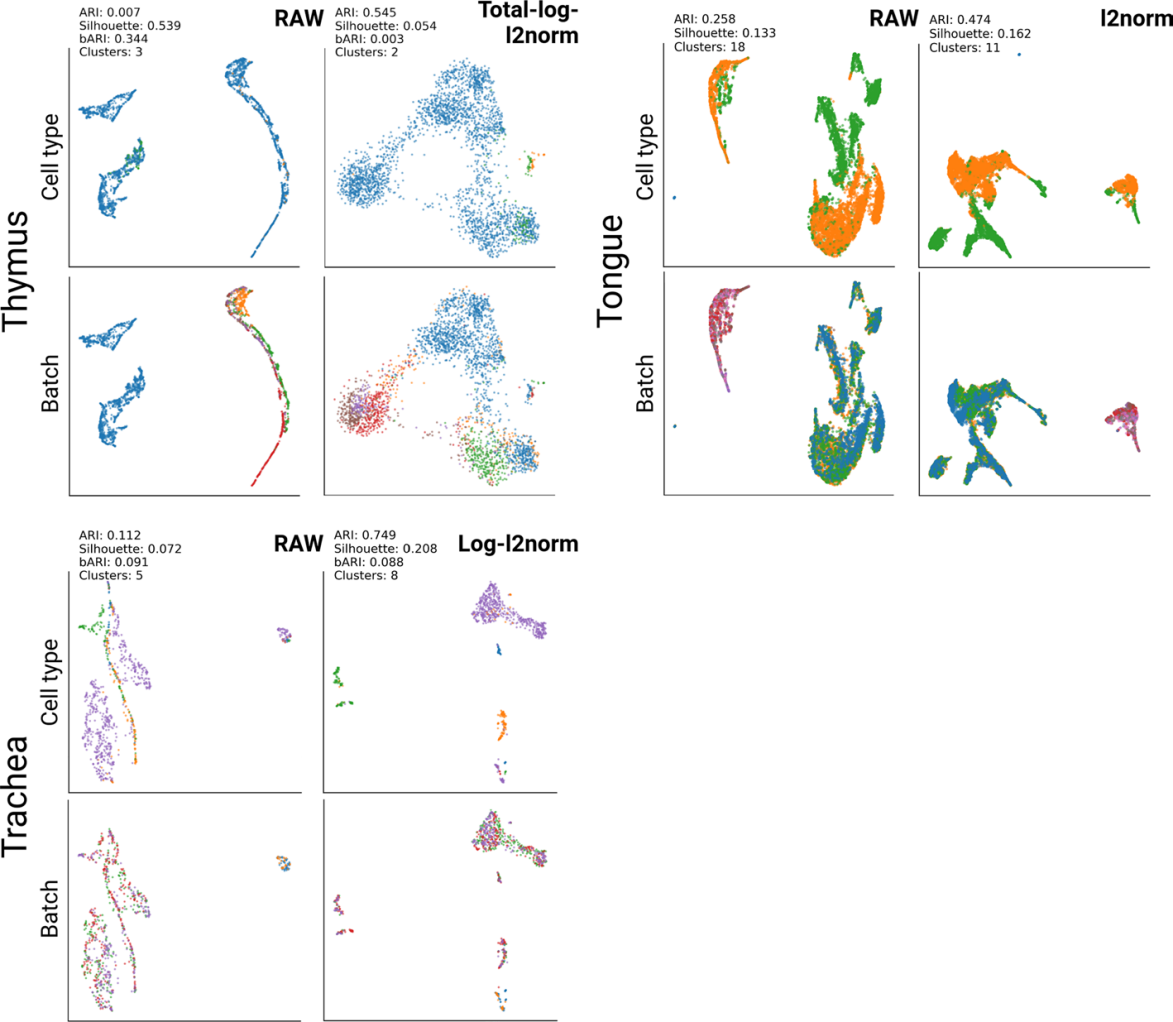
Visualization results for the Tabula Muris dataset. UMAP and DBSCAN were used for this analysis. ’RAW’ indicates the original read count data of TM. ’Best’ represents the best result out of 16 data transformation methods. The cell types and batch IDs are represented with different colors in each plot. The entire results and all plots are available in a Additional file 1

The Tabula Muris dataset consists of two different scRNA-seq datasets, SMART-Seq2 and CellRanger. Therefore, in addition to the technical heterogeneity, there is also biological heterogeneity in each dataset. Thus, we investigated the tissue pairs present in both datasets. Similar to the previous results, the data transformation methods affect the dataset integration of the TM. Depending on the method, resulting cell clusters were either based on the batch label or based on biological cell labels. Significant improvements were observed in 9 out of 11 tissue pairs of the TM dataset (see Fig. 3). The results were obtained based on the same analysis procedures, DBSCAN clustering on the low dimensional UMAP representation. In the case of the bladder dataset, mesenchymal cell (purple) and bladder cell (orange) are represented by two different clusters in ’RAW’. These clusters are strongly influenced by the batch effect and thus can be labeled by SMART-Seq2 and CellRanger. However, after data transformation with log2$\rightarrow$Minmax normalization, the cells clustered well based on the cell type labels, and the batch labels were mixed across the cluster (see Fig. 3). Accordingly, the ARI score improved from 0.347 to 0.812, and the bARI score decreased from 0.268 to $-$0.002. Similarly, in the lung dataset, the raw dataset showed batch-associated clusters. However, after transforming the scRNA-seq data with Total$\rightarrow$Log2$\rightarrow$*l*2-norm, we were able to identify cell-type label-associated clusters based on the low-dimensional representation calculated by UMAP. However, the tongue dataset showed relatively marginal batch effect correction. For instance, when performing UMAP and DBSCAN analysis, the ARI solely improved from 0.258 to 0.474 (*l2*-norm). The best results among 16 different data transformations could not find clear cell-type clusters. Full plots for each of the tissues are available in a Additional file 1 (see Methods section 5.4).

In addition to the Tabula Muris dataset, we integrated the Mouse Cell Atlas dataset, which integrates single-cell RNA sequencing datasets from mouse organs and tissues. However, the Mouse Cell Atlas dataset has a different level of cell labels compared to the other datasets. Thus the ARI and bARI are not dramatically improved. However, we observed a varying representation of cell clusters in latent space depending on the data transformation method (Data available in a Additional file 1 (See see availability of data and materials section).

#### Subtask 4: Trajectory analysis with integrated dataset

Lastly, we assessed the impact of data transformation on cell differentiation trajectory analysis. We acquired human and mouse embryo data sets that encompass cellular trajectories and applied various normalization approaches.

**Fig. 4:**
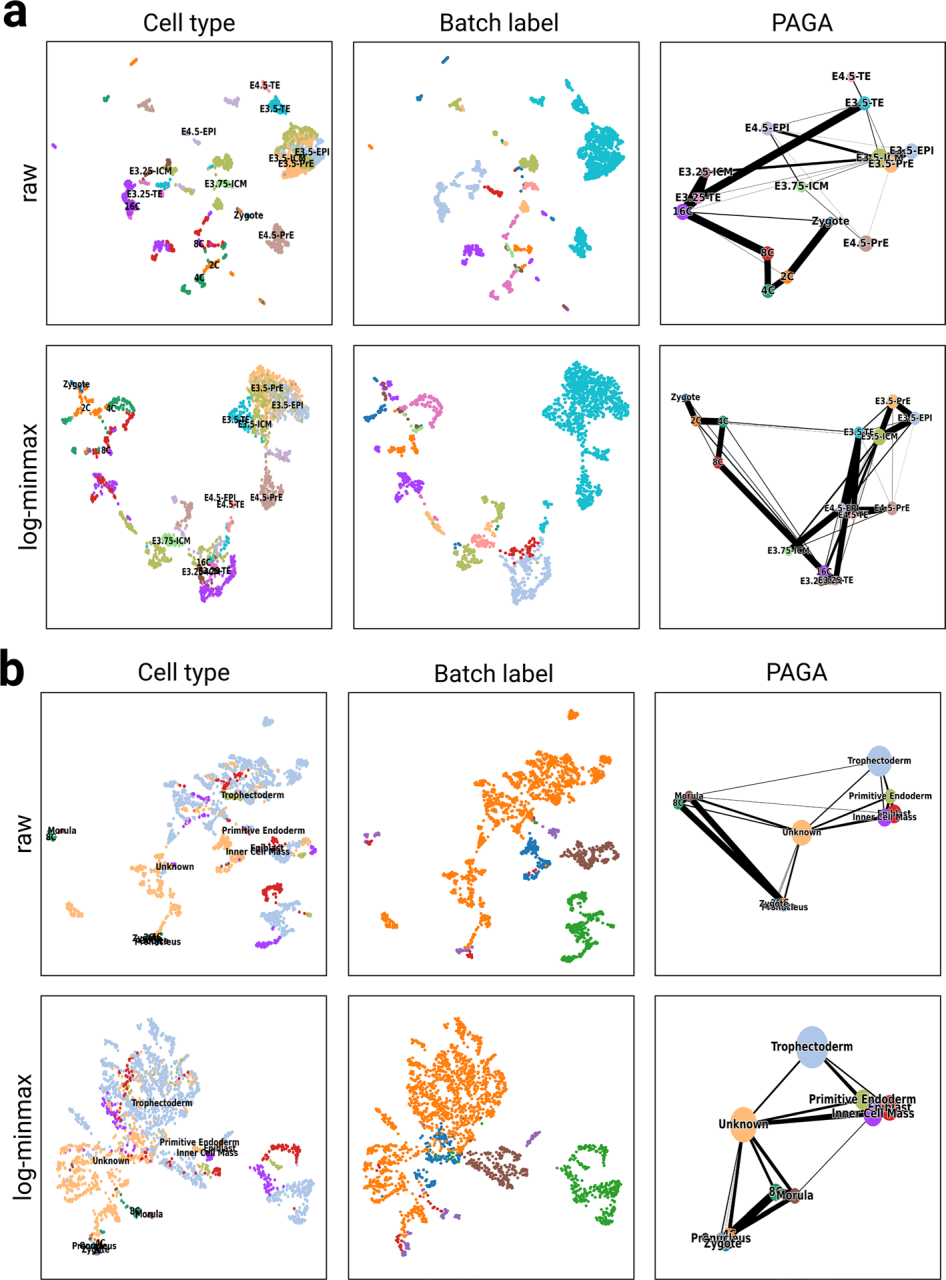
Cell differentiation trajectory analysis with multiple datasets integration. **a** Plots show the analysis results of the mouse preimplantation embryo dataset with different data transformations. **b** Plots show the analysis results of the human preimplantation embryo dataset analysis with raw and log-minmax data transformation

The initial analysis of mouse embryo data without normalization revealed an ARI score of 0.296 and a 1-bARI score of 0.338 after DBSCAN clustering on UMAP latent space. Upon log-minmax normalization of all integrated datasets, the ARI and 1-bARI scores notably increased to 0.409 and 0.535, respectively. In the pseudotime analysis of raw data, a correlation coefficient of 0.580 was observed. When dataset is normalized with the log-minmax, the correleation coefficient improved to 0.653, positioning it as the second-best trajectory result after the log transformation (s: 0.731, ARI: 0.379, 1-bARI: 0.425). The visualization depicting cell clusters and trajectory analysis conducted using PAGA can be found in Fig. 4a.

The raw human embryo data yielded an ARI score of 0.146 and a 1-bARI score of 0.226 from the DBSCAN clustering with UMAP features. Among 16 data transformations tested, the log-minmax transformation demonstrated the highest 1-bARI score of 0.938 (ARI: 0.071). The correlation coefficient for the log-minmax data transformation was 0.815, ranking it third in pseudotime analysis results. The top two pseudotime analysis outcomes were achieved with the log transformation (s: 0.823, ARI: 0.171, 1-bARI: 0.900) and log-zscore transformation (s: 0.818, ARI: 0.199, 1-bARI: 0.911). The visualization depicting cell clusters and trajectory analysis conducted using PAGA can be found in Fig. 4b.

The changes in pseudotime analysis due to the neighbor search using UMAP and different data normalization are significant because they affect the landscape of cell clusters in UMAP space. Different data normalization methods can lead to variations in the UMAP space representation of the data, as shown in previous sections. Normalization is a critical step in preprocessing scRNA-seq data, as it ensures that the gene expression values are on a comparable scale across different genes and samples. Different data transformations can affect the relative distances and relationships between cells in the UMAP space, leading to different cluster structures. This, in turn, can influence the pseudotime analysis, as the identification of clusters and the construction of pseudotime trajectories are dependent on the spatial arrangement of cells in the UMAP space.

### Impact of data transformation on the DNN features

To demonstrate a proper comparison for the model evaluation, we built various neural network models and evaluated their performance by comparing them with the above results. Here we consider the best benchmark result as a baseline for the given human pancreas dataset, we assessed the power of the DNN model.

Previously, various tools using deep neural network-based models have been developed for single-cell analysis. Moreover, autoencoder is another widely used method to extract features, reduce the dimensionality of sequencing data, and represent cell types with feature vectors. Depending on the implementation details, some tools produce a 2-dimensional vector representation like t-SNE and UMAP. Others focus on feature extraction itself and require t-SNE and UMAP as a further dimensionality reduction step for 2-dimensional representation.

We implemented the simplest neural network-based model as a feature extractor. With this DNN model task, we were able to evaluate the importance of data transformation methods in terms of ’Garbage In, Garbage Out’. At first, we tested an untrained neural network model. The simplest neural network with two fully connected layers initialized with random weights was built on the RAW as well as the best performing ’Total’ transformed single-cell RNA-seq data of the human pancreas dataset. In the latent space of the DNN trained on the ’RAW’ data, the same cell types are segregated amongst different clusters that are based on batch number. This resulted in an ARI score of 0.542 using t-SNE+DBSCAN and 0.420 using UMAP+DBSCAN with a latent size 128 and one hidden layer having a size of 1024. In contrast, the latent space of the DNN trained on the ’Total’ transformed data showed a significantly better clustering of cell types with an ARI score of 0.891 at latent vector size 128. While the randomness of the weight initialization step in every run has produced slightly different numbers, the data transformation step consistently led to a significant improvement of the model performance.

Moreover, to investigate the feature extraction power of autoencoder models, we tested autoencoder (AE) and variational autoencoder (VAE) with two layers for encoder and decoder (see "Methods" section for details). Here, we used only the Baron dataset for training and evaluated the model on the remaining entire HP dataset. Our results show that the DNN model learns to extract features about cell types only within the Baron data. If the batch effect is not mitigated by preprocessing with data transformation, the DNN model is not able to extract proper signatures for cell types in another dataset (e.g. the HP). The performance of the autoencoder without data transformation was 0.625 ARI using t-SNE+DBSCAN and 0.551 ARI using UMAP+DBSCAN (Additional file 1: figure S2). The ’Total’ transformed data showed a better performance than the ’RAW’ data even in combination with the AE and VAE DNN models. The combination of the ’Total’ transformed data with an AE model resulted in a best ARI score of 0.947 (Additional file 1: figure S3). Similarly, the best result in combination with a VAE showed an ARI of 0.943 with a latent size of 128 with a hidden layer having a size of 1024 (Additional file 1: figure S5). The overall range of performances is shown in Additional file 1: Table S3. In summary, when we compared the result with the previous analysis, using the DNN model could improve the clustering result (RAW 0.494 $\rightarrow$ 0.551 / Total 0.898 $\rightarrow$ 0.947). However, the data transformation has more impact on the clustering results.

In the next step, we used the cell labels for the evaluation of supervised dimensionality reduction methods. Therefore, we modified AE and VAE to calculate the ProtoTypical loss. Similar to the previous task, the training was conducted solely using the Baron dataset and its associate cell type labels. With the ProtoTypical loss, the impact of data transformation is also more critical than the model complexity. The results demonstrate that the use of the raw dataset leads to overfitting to the batch noise and subsequent poor cell type clustering (Additional file 1: figures S4, S6). We tested five different sizes for the latent vector from 2 to 32 while a hidden layer is fixed with a size of 1024. At the size eight, we could find convergence (Additional file 1: figure S4). While it remains to be evaluated whether more complex encoder/decoder models and more optimized parameters could result in performance improvements, our results indicate that latent space sizes between 8 and 32 are reasonable and 50 or 100 is also enough for the size of latent vector as reported in the other studies [11, 27, 40, 43].

## Discussion

The integration and analysis of single-cell RNA sequencing data have become an accessible and essential aspect of many research areas and scientific questions. Subsequently, various single-cell analysis tools emerged. The developed tools typically comprise preprocessing as well as analysis methods. The preprocessing steps can, for example, implement data transformation and de-noising, while analysis steps often include sophisticated machine learning models such as clustering and visualization by dimensionality reduction. The data transformation as the very first step of a preprocessing and analysis pipeline was not well investigated in the many benchmark papers for batch effect mitigation [17, 23, 28]. Previously, Wang et al. reported the impact of preprocessing on single-cell analysis; however, they did not cover an integrative analysis of multiple datasets where batch effects are a tremendous challenge [51]. The benchmark work by Luecken et al. tried to find an optimal preprocessing step for various tools [28]. However, they did not focus on the data normalization step.

Although we are aware that it is controversial to use the distorted t-SNE space for further analysis [85], we wanted to investigate whether this distortion enhances, mitigates, or has no effect on the analysis. The application of t-SNE by analyzing the space for meaningful information retrieval is still valid [86, 87]. In conventional single-cell analysis, Louvain or Leiden clustering is commonly used [10]. By combining dimensionality reduction techniques like t-SNE with graph-based clustering algorithms, it is possible to gain a more comprehensive understanding of complex scRNA-seq datasets and uncover meaningful structures. While they can be applied in various contexts, including high-dimensional data analysis, they are not inherently designed for dimensionality reduction like t-SNE. In this low-dimensional approach, the community detection algorithms used to understand the structure of large and complex networks, such as Leiden or Louvain, were not able to outperform those two conventional clustering algorithms. For clustering, we employed KMeans and DBSCAN algorithms.

The data transformation significantly impacts the results of downstream single-cell RNA sequencing analysis [30, 51, 88]. We conducted four integrative analysis tasks using four different datasets. Our results demonstrate that simple data transformation in low-dimensional representation analysis can effectively reduce batch effects to a similar extent as conventional batch integration methods. We demonstrated that without a complex model for batch effect mitigation, well-combined data transformation and dimensionality reduction methods show good performance in a cell-type clustering task. Additionally, we have underscored the importance of data transformation in pseudotime analysis. Ahlmann-Eltze and Huber found that a simple shifted logarithm transformation with principal component analysis showed better performance in recovering latent structure among the cells [89]. Their work underscores the efficacy of utilizing lower-dimensional embeddings derived from the transformed count matrix. This approach serves to diminish noise while enhancing fidelity. Our results highlight that lower-dimensional embeddings, after proper normalization, can reduce one of these noises, batch effects, across multiple single-cell RNA sequencing datasets. Lause et al. discussed how data transformation or scaling can affect the gene selection step and its downstream analysis. They pointed out that highly variable gene selection methods usually use their mean and variance value. In particular, they showed that the analytic Pearson residuals method works best for variable gene selections, but log-transformation also had good performance [90]. However, in our study, we excluded the variable gene selection step to retain a clear view of the impact of data transformation on the low-dimensional representation.

Lastly, we investigated the potential of DNN-based models to find batch-mitigated feature space for single-cell integration analysis. The DNN models were able to compress gene expression profiles into very small-sized vectors and made it possible to project efficiently onto a low-dimensional space for clustering and visualization. Furthermore, we aimed to check the potential of the supervised dimensionality reduction method with a ProtoTypical loss. The ProtoTypical loss allowed us to fully utilize the class label. This supervised dimensionality reduction model can be adjusted for specific research questions. Our findings suggest that in circumstances where the datasets are already well understood, ProtoTypical network models can be a good option to investigate underlying biological meanings. For example, finding novel gene markers for specific cell types. The potential of this kind of approach is also discussed in recent work [91].

Given the complexity of scRNA-seq data and the variability across datasets, it is indeed challenging to find a one-size-fits-all data normalization approach. Our results demonstrate that identifying the proper data transformation is a crucial initial step for scRNA-seq integrative analysis. To achieve this, we propose using a batch-ARI score along with the number of clusters as a metric to investigate the appropriate data transformation method for a given dataset. Researchers can utilize prior information about the data to explore improved data transformation methods for scRNA-seq analysis.

In scenarios where researchers have prior knowledge about each dataset, they can exploit this information to evaluate the integrative analysis pipeline, including the selection of suitable data transformation and integration methods. For instance, in the UMAP with DBSCAN results with the five pancreas datasets, Total-log2-Zscore, log2, and Total showed low batch-ARI scores. The Total-log2-Zscore transformation method resulted in the lowest batch-ARI score ($-$0.060) but identified four clusters, whereas the log2 transformation method found only two clusters and had a batch-ARI score of $-$0.015. In comparison, the Total transformation method, with nine clusters identified and a batch-ARI score of $-$0.011, exhibited higher clustering quality with a slightly lower batch-ARI score, indicating the preservation of the true data structure. With prior knowledge that the given pancreas datasets contain thirteen cell types, researchers can begin by employing the Total transformation, identifying nine clusters. Finding appropriate data normalization for batch integration in scRNA-seq data analysis is challenging when lacking prior knowledge of the datasets. Therefore, it is crucial to conduct individual scRNA-seq data analyses before initiating integrative analysis. This approach ensures that the data is preprocessed and normalized in a way that is suitable for subsequent batch integration. In summary, our recommendation for evaluating data transformation methods in scRNA-seq analysis, utilizing batch-ARI scores and the number of clusters, offers a data-driven approach to assess various data transformations and select the one that best aligns with researchers’ hypotheses.

## Conclusions

To the best of our knowledge, we present the first evaluation of the impact of data transformation on low-dimensional integration of single-cell RNA sequencing data. Our study demonstrates that data preprocessing is a crucial step for integrative data analysis and requires optimization for the subsequent analysis pipeline. This indicates the importance of an adequately chosen optimal data transformation method, particularly as a baseline gold standard for evaluating the performance of the subsequent analysis methodology. Furthermore, our results suggest that low-dimensional representation with proper data transformation could easily capture common gene expression signatures for cell type identification in heterogeneous batch datasets. We envision that our work will guide future integrative data analysis and also help sophisticated model development by proposing the correct baseline accuracy.

### Supplementary Information

**Additional file 1**. Supplementary figures and tables.

## Data Availability

All code, plots and datasets used in this analysis are available at the GitHub repository: https://github.com/iron-lion/scRNAseq-preprocessing-impact. All visualized results are available in https://iron-lion.github.io/scRNAseq-preprocessing-impact-doc/chapters/intro/intro.html.
