# Supplementary material for: The effect of data transformation on low-dimensional integration of single-cell RNA-seq
Source: BMC Bioinformatics. 2024 Apr 30;25:171. doi: 10.1186/s12859-024-05788-5 (PMC11059821; doi:10.1186/s12859-024-05788-5)
Supplement: Supplementary file 1 — Additional file 1. Supplementary figures and tables. [file 12859_2024_5788_MOESM1_ESM.pdf]

A Supplementary file for 'The Effect of Data Transformation on Low-Dimensional Integration of Single-Cell RNA-Seq'

Youngjun Park and Anne-Christin Hauschild

Contributing authors: anne-christin.hauschild@med.uni-goettingen.de;

Table S 1 Cell-type clustering performance comparison with the recent benchmark study.

Tools	MP	HP
Harmony ∗	0.969	0.955
Scanorama ∗	0.915	0.859
Seruat ∗	0.944	0.968
scVAE-GM ∗	0.805	NA
scVI ∗	0.932	0.759
LIGER ∗	0.914	0.911
scVI-LD ∗	0.875	0.656
scETM ∗	0.946	0.943
scETM $-\lambda$ ∗	0.851	0.474
scETM + adv ∗	0.944	0.946
$l2$-norm + UMAP+ DBSCAN	0.902	0.761
Total + UMAP+ DBSCAN	0.848	0.725
Minmax + t-SNE+ DBSCAN	0.929	NA

MP: Baron (Mouse) data
HP: Human pancreas datasets
∗ data are directly derived from the result by Zhao *et al.* [**?**]

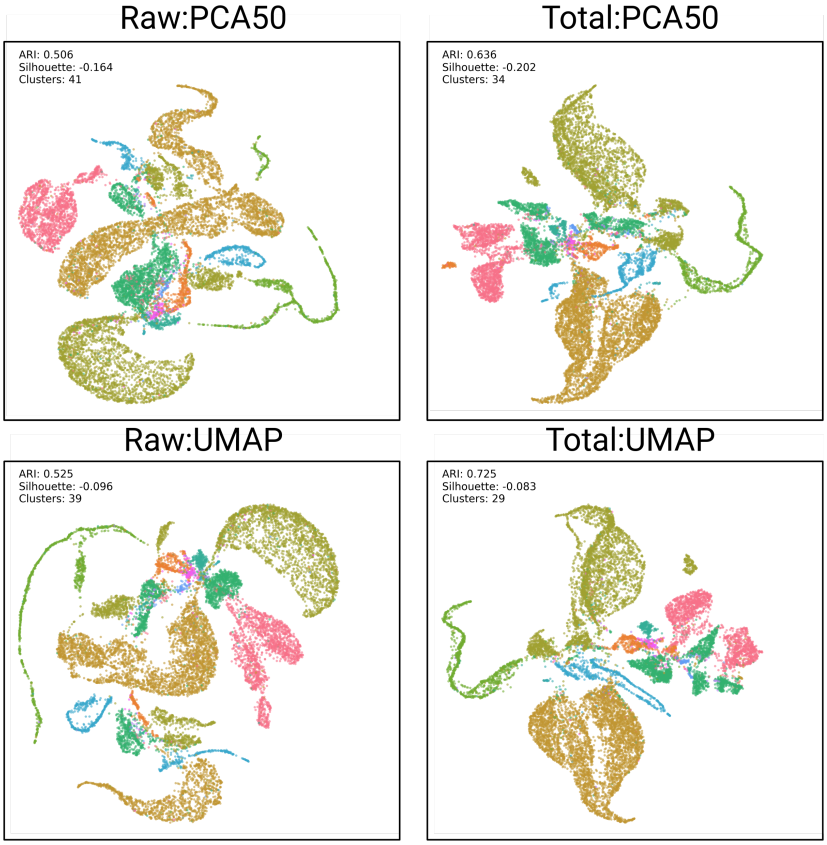

Fig. S 1 'Panc8' dataset analysis with low-dimensional representation. Human pancreas dataset is analyzed with PCA50 and UMAP-2D. The top-left plot shows a visualization result of an unintegrated dataset after dimensionality reduction with PCA50. The top-right plot shows a better clustering result with total normalized data. The ARI score is improved from 0.506 to 0.636. The bottom-left plot shows UMAP feature representation with raw data. The bottom-right plot shows better a clustering result when total normalization is applied. The ARI score is improved from 0.525 to 0.725.

Table S 2 DNN models' performance range from 10+ repeats with random seed.

	Reconstruct RAW	Total	MAML RAW	Total
AE-tSNE-KMean	0.383∼0.458	0.382∼0.511	0.315∼0.459	0.433∼0.532
AE-tSNE-DBSCAN	0.454∼0.521	0.476∼0.898	0.390∼0.615	0.606∼0.898
AE-UMAP-KMean	0.452∼0.496	0.492∼0.707	0.384∼0.507	0.686∼0.733
AE-UMAP-DBSCAN	0.299∼0.547	0.500∼0.829	0.464∼0.568	0.836∼0.947
VAE-tSNE-KMean	0.219∼0.346	0.432∼0.522	0.356∼0.468	0.454∼0.571
VAE-tSNE-DBSCAN	0.194∼0.618	0.558∼0.897	0.382∼0.578	0.520∼0.925
VAE-UMAP-KMean	0.229∼0.370	0.611∼0.785	0.419∼0.495	0.537∼0.657
VAE-UMAP-DBSCAN	0.063∼0.387	0.444∼0.894	0.438∼0.575	0.526∼0.943
Proto-tSNE-KMean	NA	NA	0.359∼0.521	0.418∼0.632
Proto-tSNE-DBSCAN	NA	NA	0.456∼0.574	0.711∼0.933
Proto-UMAP-KMean	NA	NA	0.429∼0.502	0.641∼0.737
Proto-UMAP-DBSCAN	NA	NA	0.476∼0.576	0.845∼0.907
VProto-tSNE-KMean	NA	NA	0.356∼0.488	0.454∼0.586
VProto-tSNE-DBSCAN	NA	NA	0.470∼0.597	0.587∼0.933
VProto-UMAP-KMean	NA	NA	0.484∼0.533	0.620∼0.740
VProto-UMAP-DBSCAN	NA	NA	0.494∼0.587	0.669∼0.903

Reconstruct: 20 epochs, batch size 64, lr=0.5, latent vector size 128
MAML: 200 episodes, batch/query size 5, lr=0.0005, latent vector size 128
∗ The model was trained with Baron dataset and tested with HP dataset.

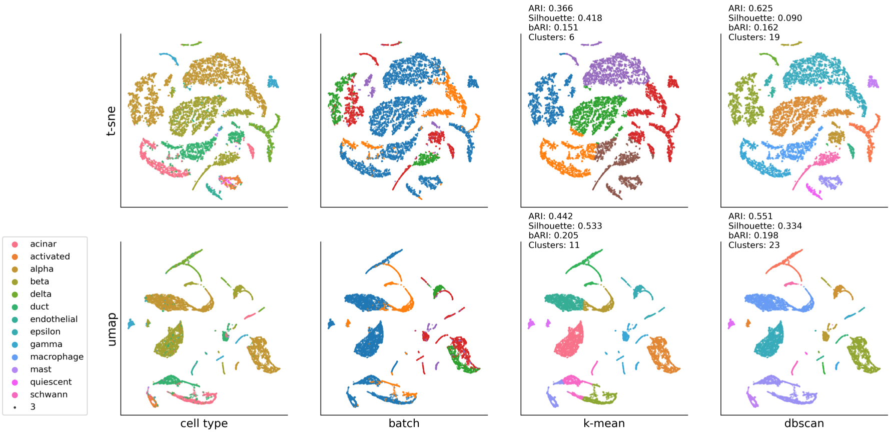

Fig. S 2 Latent representation using Autoencoder without data transformation. The model was trained by MAML 200 episodes with the Baron dataset and evaluated on HP datasets. Human pancreas datasets were not processed. Latent vector size is set to 128.

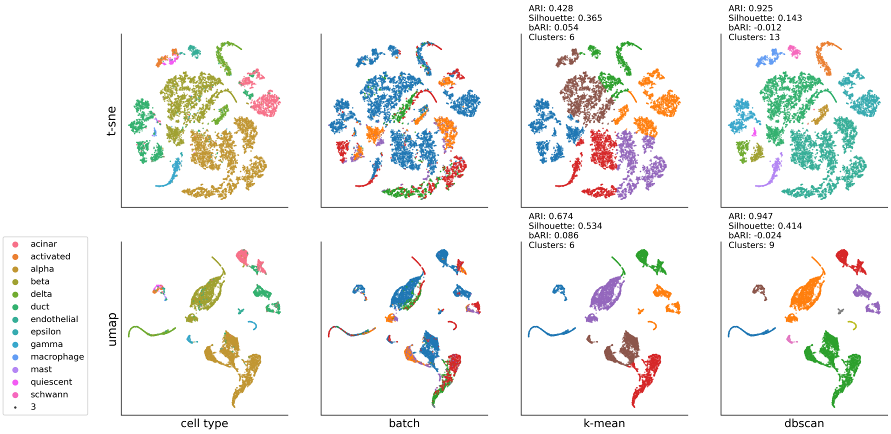

Fig. S 3 Latent representation using Autoencoder with Total data transformation. The model was trained by MAML 200 episodes with the Baron dataset and evaluated on the HP datasets. Human pancreas datasets were transformed with Total. Latent vector size is set to 128.

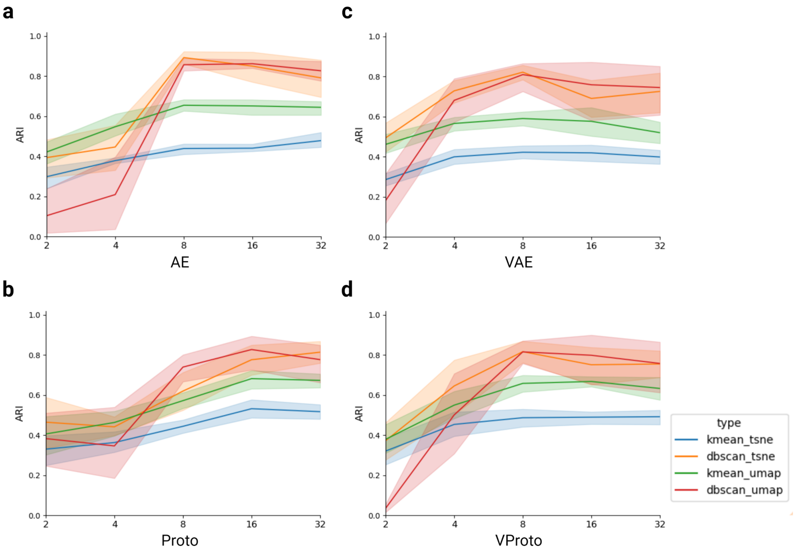

Fig. S 4 Evaluation of the performance of deep neural network models with varying sizes of the latent vector. Shown ARI scores are obtained after 200 episodes of MAML training with Baron (Human) datasets. All human pancreas datasets are transformed with Total. The x-axis corresponds to the size of the latent vector. The colored area represents a 95% confidence interval with more than 6+ repeats of training and evaluations. Abbreviation: Autoencoder (AE), Variational Autoencoder (VAE), ProtoTypical Network on AE backbone (Proto), ProtoTypical Network on VAE backbone (VProto).

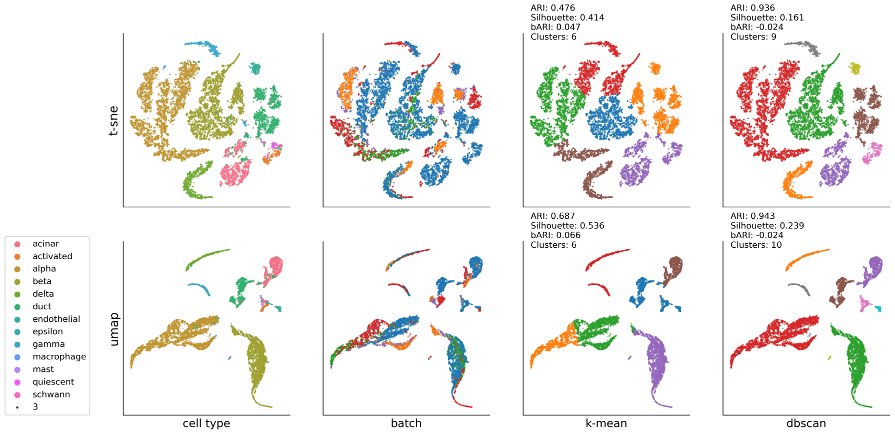

Fig. S 5 Latent representation using Variational Autoencoder with Total data transformation. The model was trained by MAML 200 episodes with the Baron dataset and evaluated on the HP datasets. Human pancreas dataset is transformed with Total method. Latent vector size is set to 128.

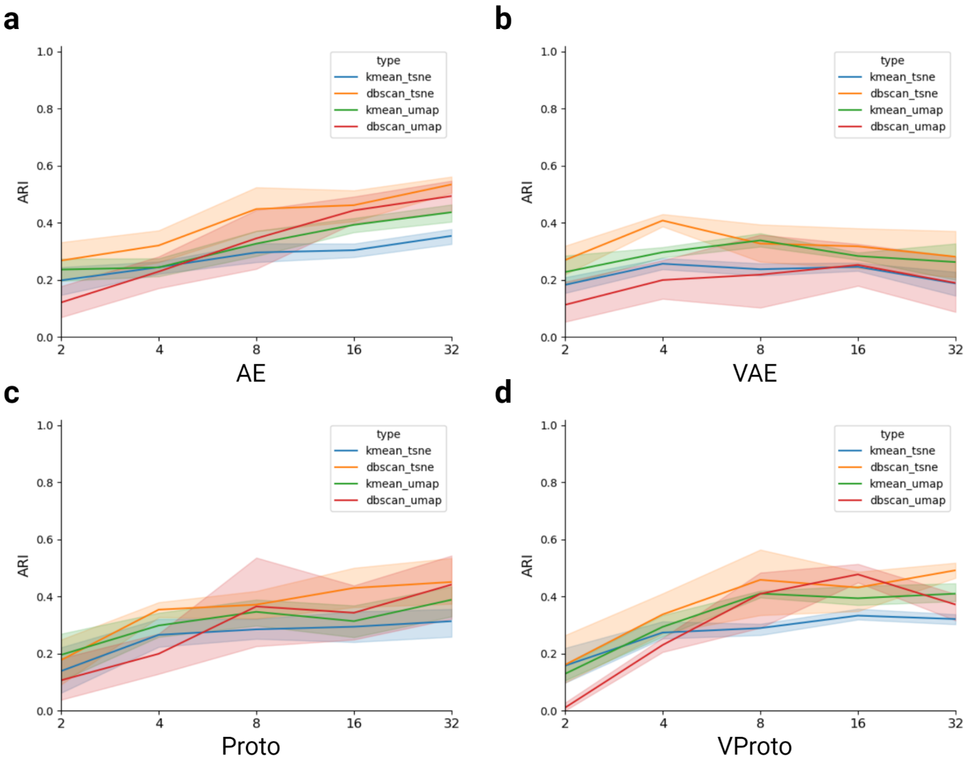

Fig. S 6 The performance of deep neural networks models with different size of latent vectors. When RAW expression count is used, the AE and VAE models showed relatively poor ARI compared to Total transformed data (Figure ??). All models were trained with 200 episodes of MAML training with Baron datasets and evaluated.
